# Ischemic Stroke After Tumor Resection in a Patient With Glioblastoma Multiforme

**DOI:** 10.7759/cureus.13232

**Published:** 2021-02-08

**Authors:** Sara Soliman, Medhat Ghaly

**Affiliations:** 1 Internal Medicine, Yale-Waterbury Internal Medicine Residency Program, Waterbury, USA; 2 Internal Medicine, Yale School of Medicine, New Haven, USA

**Keywords:** glioma, hemorrhagic stroke, glioblastoma multiforme, thrombolysis

## Abstract

Glioblastoma multiforme (GM) is the most common type of aggressive malignant glioma in the brain or spinal cord and represents 15% of all primary brain tumors among adults. Although ischemic strokes in the setting of an underlying glioma is a rare occurrence, its diagnosis is usually challenging due to the overlapping neurological manifestations with the underlying brain tumor. We report a case of a 58-year-old white male who presented with subacute worsening symptoms of expressive aphasia with focal neurological symptoms, including right-sided extremity motor weakness and intermittent vision spots. Magnetic resonance imaging (MRI) of brain revealed a large 9.5 cm infiltrating mass in the left frontal and temporal lobes, strongly indicative of a primary glioma. The patient underwent resection to confirm diagnosis and remove part of the tumor mass. Pathological examination revealed GM. Expressive aphasia was markedly improved following the surgery; however, on postoperative day 3, the patient developed acute onset of right-sided weakness and sensory deficit. MRI revealed acute left posterior, frontal, and parietal infarct. Unfortunately, recent brain surgery would not allow for intravenous thrombolysis, and, therefore, he was discharged with a plan for outpatient radiation treatment and oral temozolomide chemotherapy.

## Introduction

Glioblastoma multiforme (GM) is the most common and aggressive primary brain tumor with poor prognosis and resistance to conventional chemotherapy and radiotherapy [1], with approximate postoperative survival time of less than 15 months [2]. The focal neurological symptoms associated with glioma are primarily due to direct brain infiltration, mass effect, and vasogenic edema [3]. Hemorrhagic strokes are common occurrence in glioma patients; nonetheless, ischemic infarcts are extremely rare with only few reported cases [2,4,5]. Due to the similar neurological deficits between an ischemic stroke and the underlying glioma, stroke diagnosis is usually clinically challenging, which could result in late intervention and poor management. Here we present a case of GM with postoperative ischemic stroke in a 58-year-old male. Ischemic stroke should be considered in the differential diagnosis of GM, especially after resection surgery. Given the poor prognosis and perilous complications associated with ischemic stroke and underlying glioma, early diagnosis and prompt management are of utter importance.

## Case presentation

**Figure 1:**
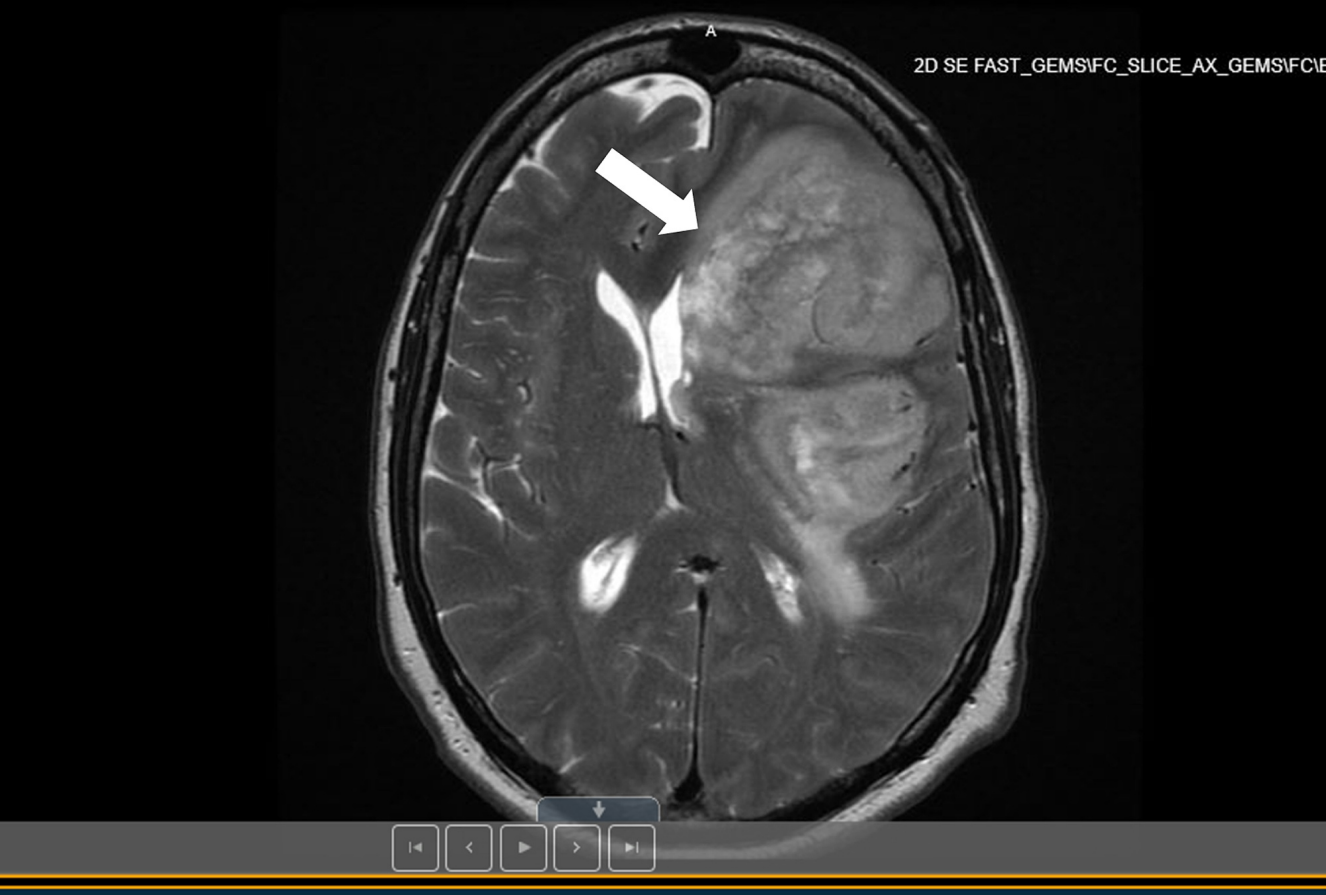
MRI of the brain reveals a large 9.5 cm infiltrating intra-axial mass in the left frontal and temporal lobe with 13 mm midline shift from left to right (indicated with a white arrow).

A 58-year-old white male with history of controlled hypertension currently on amlodipine presented with a six-month history of subacute neurologic deficits, including expressive aphasia, right-sided extremity motor weakness, intermittent vision spots, and severe headache. Six weeks prior to presentation, he noted progressive worsening of his symptoms. Clinical examination was significant of expressive aphasia and word-finding difficulty, but no motor or sensory deficits. Magnetic resonance imaging (MRI) of brain revealed a large intra-axial mass infiltrating the left frontal and temporal lobes, measuring 9.5 cm, strongly indicative of primary glioma (Figure 1).

 

**Figure 2:**
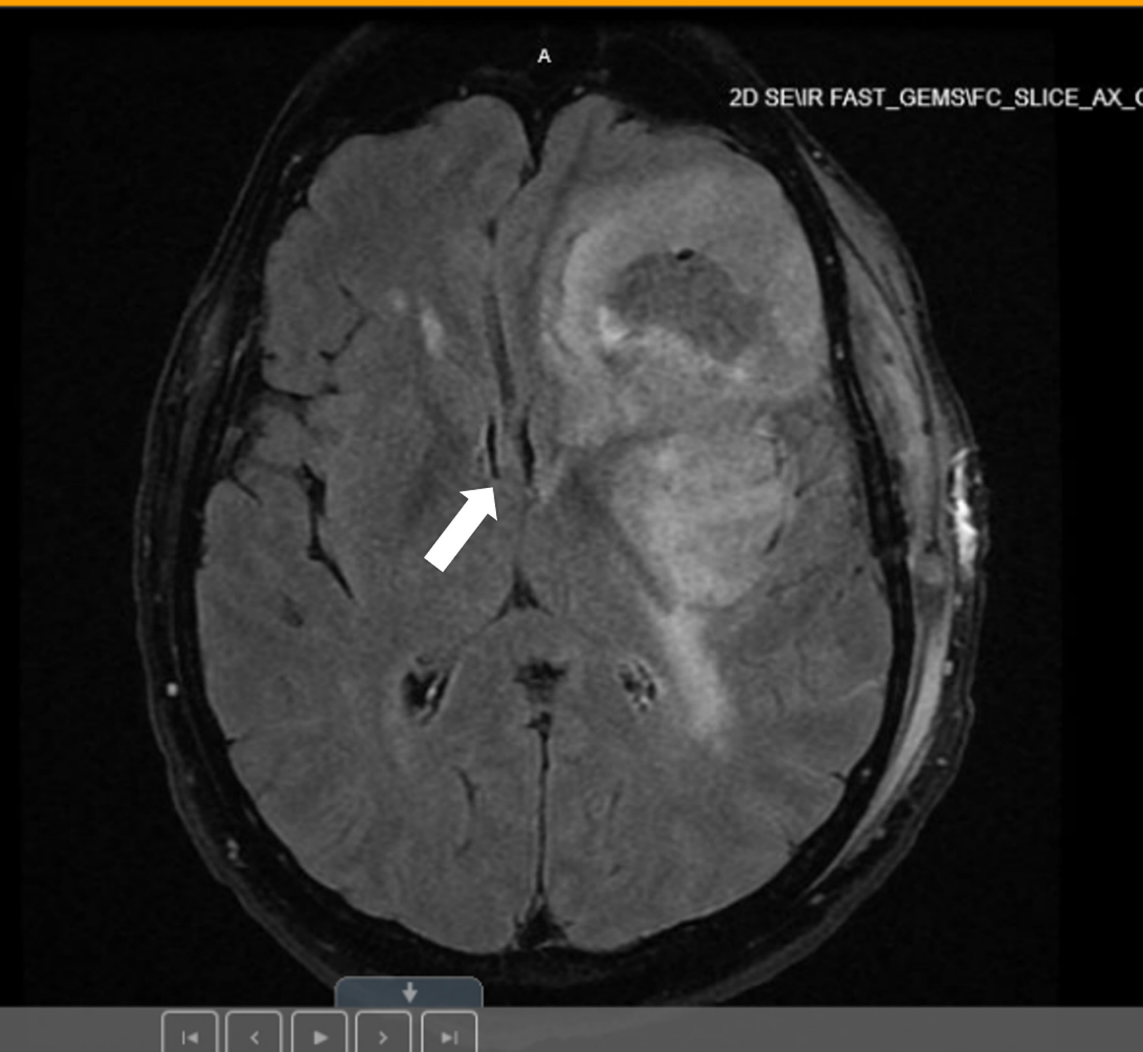
MRI of the brain demonstrates post partial resection of the large left frontotemporal tumor, with decreased peripheral contrast enhancement and decreased mass effect, resulting in diminished midline shift from left to right (denoted by a white arrow), now measuring 9 mm, compared to 13 mm preoperatively.

He was started on 4 mg intravenous (IV) dexamethasone every 6 hours to reduce mass effect and vasogenic edema. Levetiracetam (1 g/day) was also given for seizure prophylaxis. Subsequently, he underwent left craniotomy and resection of left frontal tumor. Postoperative MRI revealed partial resection of the tumor with decreased mass effect, resulting in diminished midline shift to 9 mm compared to 13 mm preoperatively (Figure 2). At this time, there was no indication of acute ischemic injury. Pathological examination confirmed GM.

 

**Figure 3:**
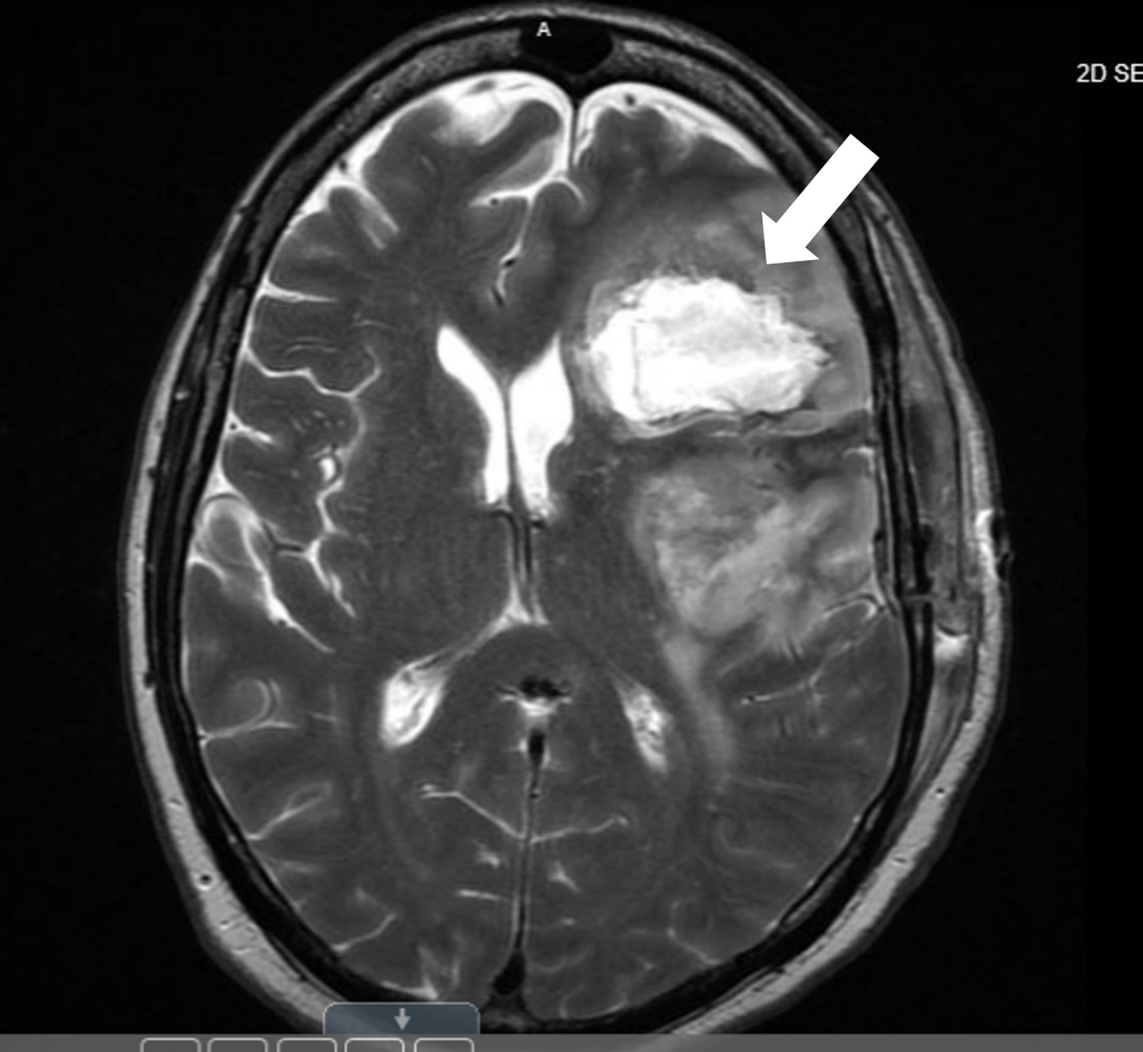
MRI of the brain reveals acute left posterior, frontal, and parietal infarct status post a recent partial resection of the left frontotemporal lobe tumor (indicated with a white arrow)

Postoperatively, there was initial worsening of neurological deficits, specifically expressive aphasia, followed by significant improvement. On postoperative day 3, the patient developed acute onset of right-sided hemiparesis with marked aphasia. MRI of brain showed acute left posterior, frontal, and parietal infarct (Figure 3). Given the recent brain surgery and underlying malignancy, the patient was not candidate for IV thrombosis with plasminogen tissue activator (tPA). Patient was discharged with a plan to start Stupp protocol of temozolomide and concomitant radiotherapy [6].

## Discussion

When compared to the various types of brain tumors, glioma is associated with the highest mortality rate, a rarely curable form, resistant to chemotherapy and radiotherapy, and has unfavorable prognosis [7]. Additionally, glioma and other brain tumors are risk factors for acute ischemic infarction and the opposite was also shown to be true [8,9]. It has been estimated that one in seven to eight patients with ischemic stroke have a known or hidden cancer [7]. Ischemic stroke patients typically develop brain tumor, mostly glioma, due to changes imposed by ischemia and hypoxia on the functional and metabolic state of the cell [9]. On the other hand, proliferating cell mass, metastasis, and general prothrombotic tendency associated with tumor increase the risk of developing ischemic infarcts [7].

There are several proposed mechanisms explaining the reasons of co-occurrence of ischemic infarct with brain tumors, particularly glioma. The most prevalent mechanism highlights the predisposition of both conditions to hypoxia [10-12]. For instance, cerebral ischemia causes obstruction of blood vessels and predisposes to hypoxia [12], whereas highly proliferating malignant mass has hypoxic core due to the increased demand of oxygen by the rapidly dividing cells [13]. Other potential mechanisms were proposed to be involved in the interplay between the two processes, such as astrocyte activation [14], reactive gliosis [15], angiogenesis, and other changes in tumor microenvironment [16], which are all primarily triggered by the cerebral ischemia as a consequence of glioma development [7]. Furthermore, the repeated resection procedures during glioma management increase the risk of ischemic injuries [7-10]. In a relevant study of 66 patients with ischemic stroke and underlying brain tumor, half of the population developed ischemic infarction as an acute operative complication [4]. Treatment modalities of glioma were also demonstrated to pose a risk for ischemic strokes. Remarkably, radiation-induced vasculopathy accelerates atherosclerosis and increases the risk of ischemic strokes, especially when combined with anti-angiogenic chemotherapy. Higher incidence of ischemic stroke and intracranial hemorrhage was also shown to be associated with the use of the anti-angiogenic agent bevacizumab as an adjuvant to radiotherapy in the treatment of recurrent glioma [17].

In addition to the tumor-related mechanism that could be involved in the development of stroke in glioma patients, conventional stroke mechanisms could also play a role in stroke etiology [10]. Therefore, there has been a controversy in stroke characteristics in tumor patients and a clinical challenge in the early diagnosis and management of the associated infarction. Given the overlapping neurological manifestations between glioma and stroke, MRI proved to be the best modality to reveal ischemia in the setting of glioblastoma as tumor cells will probably mask any changes on computed tomography (CT) [5].

Management of ischemic stroke in the setting of underlying brain tumor was also found to be clinically challenging. Although some studies recommended the use of tPA, tumor complications usually impose constraints on the thrombolysis procedure [18]. The presence of an intracranial neoplasm is generally considered a contraindication to IV thrombolysis [19], and hence very limited data exist on the use of thrombolysis in the management of stroke patients with brain tumors [20]. A relevant study reported that one of two glioblastoma patients who received thrombolysis developed intracranial hemorrhage, which is likely due to the higher propensity associated with glioma for spontaneous bleeding [20]. It is also worth mentioning that the diagnosis of both cases with glioblastoma in this particular case was not confirmed at the time of presentation, and seizures were attributed to cerebral ischemia, and thrombolysis was, therefore, the first choice in management of the acute ischemia.

Due to the clinical challenges in the early diagnosis of ischemic stroke in glioma patients, diagnosis is likely to be delayed beyond the thrombolysis window. Nevertheless, and in case of early finding of stroke, it will be important to consider the potential benefit of thrombolysis in reducing death or major disability, and at the same time weighing the possible risk of developing hemorrhage and other complications, especially if contradictions are inevitable due to the underlying tumor.

## Conclusions

We present a rare case of ischemic stroke in the setting of an underlying GM. Diagnosis and management of such cases can be challenging due to the overlapping clinical manifestations. Infarctions are likely to develop as an acute postoperative complication of resection. Physicians should always consider the possibility of postoperative stroke when examining MRI after surgery. Weighing benefits and risks of thrombolysis in stroke treatment is crucial due to the inevitable risk of intracranial bleeding. Early diagnosis and prompt intervention are of paramount importance due to the detrimental complications and poor prognosis of cerebral ischemia.
